# Draft Genome Sequences of Three Multiantibiotic-Resistant *Campylobacter jejuni* Strains (2865, 2868, and 2871) Isolated from Poultry at Retail Outlets in Malaysia

**DOI:** 10.1128/genomeA.00331-16

**Published:** 2016-05-05

**Authors:** Amy Huei Teen Teh, Sui Mae Lee, Gary A. Dykes

**Affiliations:** a School of Science, Monash University, Selangor Darul Ehsan, Malaysia; b School of Public Health, Curtin University, Western Australia, Australia

## Abstract

*Campylobacter jejuni* is a frequent cause of human bacterial gastrointestinal foodborne disease worldwide. Antibiotic resistance in this species is of public health concern. The draft genome sequences of three multiantibiotic-resistant *C. jejuni* strains (2865, 2868, and 2871) isolated from poultry at retail outlets in Malaysia are presented here.

## GENOME ANNOUNCEMENT

*Campylobacter jejuni* is one of the leading causes of bacterial gastrointestinal foodborne disease worldwide (1). It is commonly found in the gastrointestinal tract of poultry, and the consumption of undercooked poultry is the main source of human *Campylobacter* infections (2, 3). *C. jejuni* is fastidious in its growth and survival requirements. The ability of these bacteria to form biofilms has been suggested to contribute to their survival in the environment. Widespread use of antibiotics to treat campylobacteriosis has led to the emergence of antibiotic-resistant *Campylobacter* strains (4, 5). Previous studies have shown that there is a relationship between antibiotic resistance genes and the ability of bacterial strains to form biofilms (6–9). No work, however, has been reported on the relationship between the acquisition of antibiotic resistance genes and the ability of *C. jejuni* strains to form biofilms.

Three *C. jejuni* strains, designated 2865, 2868, and 2871, isolated from poultry obtained from retail outlets in Malaysia (10), were sequenced. Genome sequencing of these strains was performed using the Illumina MiSeq benchtop sequencer (250-bp paired-end reads). The raw reads generated were trimmed and assembled *de novo* using CLC Genomics Workbench 7.0 (CLC bio, Denmark). The draft genome of *C. jejuni* 2865 was assembled into 69 contigs, with a 30.2% G+C content and an accumulated length of 1,821,463 bp (*N*_50_, 83,391 bp). The draft genome of *C. jejuni* 2868 was assembled into 111 contigs, with a 30.3% G+C content and an accumulated length of 1,742,310 bp (*N*_50_, 35,675 bp). The draft genome of *C. jejuni* 2871 was assembled into 31 contigs, with a 30.4% G+C content and an accumulated length of 1,654,937 bp (*N*_50_, 180,958 bp).

Using the NCBI Prokaryotic Genome Annotation Pipeline (PGAP) and Rapid Annotations using Subsystems Technology (RAST), 1,811 coding sequences (CDSs), 3 rRNAs, and 40 tRNAs were annotated for *C. jejuni* strain 2865, while 1,701 CDSs, 4 rRNAs, and 40 tRNAs were annotated for *C. jejuni* strain 2868, and 1,650 CDSs, 3 rRNAs, and 40 tRNAs were annotated for *C. jejuni* strain 2871.

Genes related to biofilm formation processes, such as adhesion (*cadF* and *peb*) (11, 12), motility (*flgE2* and *flaAB*) (13, 14), capsular polysaccharide synthesis (*kpsM, kpsE*, and *waaF*) (15–17), and stress response (*csrA*) (18), are present in all strains. Several genes involved in resistance to antibiotics were identified in all strains, including those coding for tetracycline resistance, fluoroquinolone resistance genes, and beta-lactamases, as well as genes involved in multidrug resistance efflux pumps. Some of these genes, including the tetracycline resistance *tet*(O) gene, are present in all three strains but absent in reference strains, such as *C. jejuni* NCTC 11168. The presence of these genes might affect the ability of these three strains to form biofilms.

### Nucleotide sequence accession numbers.

The whole-genome shotgun projects of *C. jejuni* strains 2865, 2868, and 2871 have been deposited at DDBJ/EMBL/GenBank under the accession numbers LLWL00000000, LLWM00000000, and LLWN00000000, respectively. The versions described in this paper are versions LLWL00000000, LLWM00000000, and LLWN00000000.
